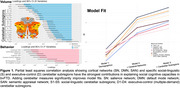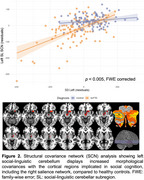# Cerebellar degeneration and its integrative role in brain networks for social cognition in early‐stage bvFTD

**DOI:** 10.1002/alz70857_099454

**Published:** 2025-12-24

**Authors:** Yu Chen, Electra Chatzidimitriou, Howard J. Rosen, William W. Seeley, Maria Luisa Gorno Tempini, Joel H Kramer, Bruce L. Miller, Katherine P Rankin

**Affiliations:** ^1^ Department of Neurology, Memory and Aging Center, University of California San Francisco, San Francisco, CA, USA; ^2^ Aristotle University of Thessaloniki, Thessaloniki, Greece; ^3^ Dyslexia Center, University of California San Francisco, San Francisco, CA, USA

## Abstract

**Background:**

Social cognitive dysfunction manifests early in behavioral variant frontotemporal dementia (bvFTD), causing significant distress and burden for caregivers. Research on impaired social cognition in bvFTD has traditionally focused on cortical networks. Emerging evidence highlights cerebellar changes associated with cognitive and neuropsychiatric symptoms in bvFTD. Notable, these changes occur at early disease stages and are accompanied by social cognitive deficits. However, the impact of cerebellar degeneration on social cognition and its relation to cortical networks in bvFTD remains unknown.

**Method:**

A total of 109 individuals with bvFTD and 100 healthy controls were evaluated across three social domains: **Theory of Mind (ToM)** (The Awareness of Social Inference test—Social Inference–Enriched, Cognitive Emotional Perspective Taking Test), **emotion reading** (Dynamic Affect Recognition Test, Comprehensive Affect Testing System), and **real‐life empathy** (Revised Self‐Monitoring Scale, Interpersonal Reactivity Index). Cerebellar subregion volumes associated with social‐linguistic and executive‐control functions, as defined by a precision cerebellar mapping atlas, were extracted from participants’ earliest structural MRIs. Region‐of‐interest‐based partial least squares correlation and voxel‐wise structural covariance network analyses were conducted to examine the contributions of cerebellar subregion volumes to social cognition and their relationship with cortical integrity.

**Result:**

Individuals with early‐stage bvFTD (Global Clinical Dementia Rating=1.1±0.6) performed worse on all assessments than healthy controls (*p* < 0.05). Significant volume loss was observed in cerebellar subregions (*p* < 0.05), including bilateral Crus I, Crus II, and lobules VIIb, which contributed to all social domains, particularly ToM and emotion reading. The most influential cerebellar subregions, the left Crus I and II, exhibited increased morphological covariance with cortical regions implicated in social cognition, including the right salience network, compared to controls (*p^FWE^* < 0.005). Incorporating cerebellar measures alongside cortical network volumes (salience, semantic appraisal, and default mode networks) significantly improved the predictive power of brain‐behavior models.

**Conclusion:**

These results demonstrate that in early‐stage bvFTD, cerebellar involvement and its interaction with cerebral network regions may play a more important role in social cognition than previously appreciated. Specifically, posterior cerebellar subregions significantly contribute to social cognitive dysfunction in bvFTD. The observed morphological covariance between cerebellar subregions and cortical social network regions underscores the cerebellum's integrative role within larger brain networks.